# The Transaxial Orientation Is Superior to Both the Short Axis and Horizontal Long Axis Orientations for Determining Right Ventricular Volume and Ejection Fraction Using Simpson's Method with Cardiac Magnetic Resonance

**DOI:** 10.1155/2013/268697

**Published:** 2013-04-14

**Authors:** Michael K. Atalay, Kevin J. Chang, David J. Grand, Shawn Haji-Momenian, Jason T. Machan, Florence H. Sheehan

**Affiliations:** ^1^Department of Diagnostic Imaging, Rhode Island Hospital, Warren Alpert School of Medicine of Brown University, 593 Eddy Street, Providence, RI 02903, USA; ^2^Association of Alexandria Radiologists, 4660 Kenmore Avenue, Suite 525, Alexandria, VA 22304, USA; ^3^Departments of Orthopaedics and Surgery, Warren Alpert School of Medicine of Brown University, Providence, RI 02903, USA; ^4^Biostatistics Research, Rhode Island Hospital, Providence, RI 02903, USA; ^5^Department of Medicine (Cardiology), University of Washington, Box 356422, Seattle, WA 98195, USA

## Abstract

We sought to determine which of the three orientations is the most reliable and accurate for quantifying right ventricular (RV) volume and ejection fraction (EF) by cardiac magnetic resonance using Simpson's method. We studied 20 patients using short axis (SA), transaxial (TA), and horizontal long axis (HLA) orientations. Three readers independently traced RV endocardial contours at end-diastole and end-systole for each orientation. End-diastolic volumes (EDVs), end-systolic volumes (ESVs), and EF were calculated and compared with the 3D piecewise smooth subdivision surface (PSSS) method. The intraclass correlation coefficients among the 3 readers for EDV, ESV, and EF were 0.92, 0.82, and 0.42, respectively, for SA, 0.95, 0.92, and 0.67 for TA, and 0.85, 0.93, and 0.69 for HLA. For mean data there was no significant difference between TA and PSSS for EDV (−2.6%, 95% CI: −8.2 to 3.3%), ESV (−5.9%, −15.2 to 4.5%), and EF (1.7%, −1.5 to 4.9%). HLA was accurate for ESV (−8.9%, −18.5 to 1.8%) and EF (−0.7%, −3.8 to 2.5%) but significantly underestimated EDV (−9.8, −16.6 to −2.4%). SA was accurate for EDV (0.5%, −6.0 to 7.5%) but overestimated ESV (10.5%, 0.1 to 21.9%) and had poor interrater reliability for EF. *Conclusions*. The TA orientation provides the most reliable and accurate measures of EDV, ESV, and EF.

## 1. Introduction

Right ventricular (RV) dysfunction can occur in several congenital and acquired disease states, and the importance of evaluating RV size and function has become increasingly evident [1, 2]. Two-dimensional echocardiography and nuclear techniques are used in clinical practice to qualitatively survey RV size and function [3, 4]. Due chiefly to the complex shape, thin wall, and substernal location of the RV, these methods are limited in their ability to accurately assess morphology and function. It has become generally accepted that MRI, with its high temporal, spatial, and contrast resolution, provides the most comprehensive and robust assessment of the RV [5–7]. However, because of its complex shape and associated problems with contour delineation and partial volume averaging, there is no clearly preferred axis for RV volumetric analysis using Simpson's method of slice summation. Although stacked short axis (SA) slices obtained for LV evaluation are routinely available in clinical cardiac magnetic resonance (CMR) and can be used to calculate RV volumes and ejection fraction (EF) [7, 8], it has recently been suggested that quantitative assessment of RV chamber volumes is more reliable using the transaxial (TA) orientation [9, 10]. 

A third potentially appealing orientation for evaluating the RV by Simpson's method is the horizontal long axis (HLA). Like the SA, the HLA is a “natural” cardiac imaging axis that depends on the relative cardiac chamber positioning and not on the standard body axes (sagittal, coronal, and transaxial). Moreover, it generally provides clear visualization of the tricuspid valve plane and the RV free wall. These features may improve endocardial contour delineation using Simpson's method. To our knowledge, use of this axis for RV volumetric analysis has not been previously reported in the literature.

Prior studies comparing TA and SA orientations lacked a validated, gold standard for determining the accuracy of measurements. The objective of this paper has been to determine which of these three orientations is the most reliable between three different experienced readers and which orientation is the most accurate when compared to the piecewise smooth subdivision surface (PSSS) method which is the only method that has been validated for accuracy in reproducing the 3-dimensional morphology of cardiac ventricles as well as for quantifying ventricular volume [11, 12].

## 2. Materials and Methods

Our hospital's Institutional Review Board (IRB) approved the design of this prospective study, and all data were handled in compliance with the Health Insurance Portability and Accountability Act (HIPAA).

### 2.1. Study Participants

Twenty consecutive patients who were referred for a clinically indicated CMR examination were enrolled (Table 1). All subjects provided informed consent. The mean age was 45 ± 19 years (standard deviation) (median age; 48 years; age range: 18–81 years). The average height was 1.7 ± 0.1 m and average weight 82 ± 24 kg. There were 10 men. 

### 2.2. MRI Imaging Protocol

**Figure 1:**
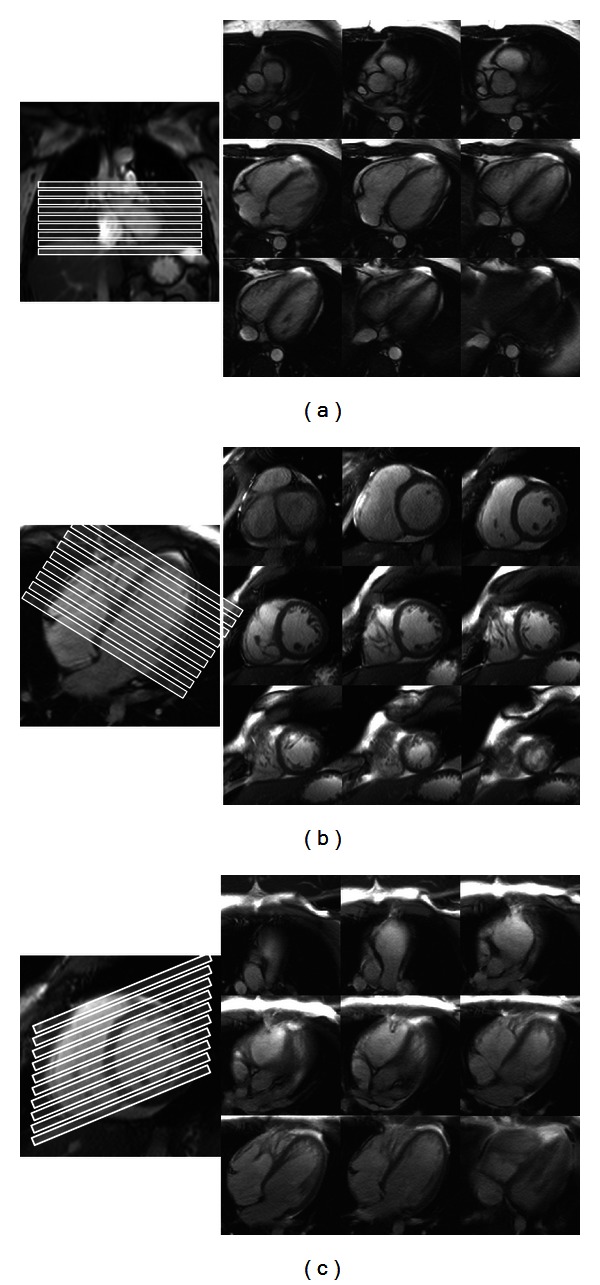
Stacked cine SSFP end-diastolic images through the heart acquired in (a) TA orientation with a coronal slice reference image, (b) SA orientation with an HLA reference image, and (c) HLA orientation with a midcavity SA reference image. Contours—not shown—were drawn in a manner similar to that depicted by Alfakih et al. [9].

**Figure 2:**
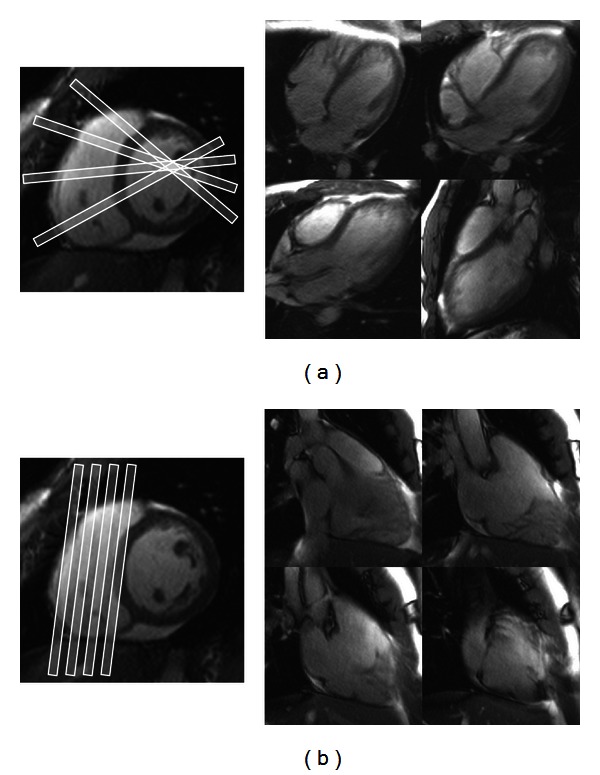
Additional cine SSFP slices were used for the PSSS method. These included (a) 4 long axis views through the RV, rotated around the center of the LV cavity at the midcavity short axis level, and (b) 4 stacked two-chamber right heart views. An SA reference image is shown.

*Axes.* All patients underwent MR imaging protocols appropriate for their clinical condition. Additional imaging relevant to this study was integrated into each examination and preceded contrast administration in cases where contrast was clinically warranted. Stacked cine steady-state free precession (SSFP) slices were obtained in SA, TA, and HLA orientations (Figure 1). The number of slices obtained in any particular orientation was variable between patients, but in all cases complete RV volumetric coverage was ensured. For the PSSS method, which is described in detail below, 3 or 4 stacked two-chamber right heart slices (RV inflow/outflow) were also obtained, as well as 4 long axis slices through the RV, and rotated around the center of the LV cavity at the midcavity short axis level (Figure 2). These additional slices were obtained in random order with the three principle orientations.

*Scan Details.* All studies were performed on a 1.5-T MRI Siemens Symphony scanner (Siemens Medical Systems, Malvern, PA, USA) equipped with Quantum gradients. Subjects were imaged in the supine position using a six-element phased-array chest coil. All cine imaging was conducted using SSFP (echo time 1.4–2.3 ms; repetition time 48–53 ms; flip angle 40–45°; FOV 26–35 cm; matrix 156 × 192) with retrospective ECG gating. Data were acquired during end-inspiration. In all cases, slice thickness was 8 mm, and slice separation was 2 mm. Twenty-five cardiac phases were reconstructed for each cine loop. 

### 2.3. Data Analysis

*Simpson's Method. *Three board certified radiologists with 1–5 years of CMR experience independently analyzed each of the data sets. Volume error due to frame selection and intraobserver variability lie within the range of interobserver variability [13–15]. Therefore end-diastolic (ED) and end-systolic (ES) phases for each patient were predetermined to focus on interobserver variability in interpreting images for analysis by Simpson's method. Image analysis was conducted offline on commercially available software (Argus; Siemens Medical Systems). Each reader manually contoured the RV endocardium at ED and ES for each patient, in each of the three principle orientations (ordered randomly). RV trabeculation and papillary muscles were considered to be part of the blood pool. The analysis software required that all contours be drawn as closed loops. For the TA and HLA orientations, the RV outflow tract (RVOT) was traced up to the level of the pulmonary valve cusps. Near the tricuspid valve, a straight line was drawn across the valve plane. Reader discretion determined the inferior-most slices to include and how to trace the contours in these slices. For the SA, readers independently decided the basal- and apical-most slices to include. Again, the RVOT was traced up to the pulmonary cusps. In general, partial volume averaging effects were handled on a case-by-case basis in a manner that was felt to be most appropriate by each reader. Readers were blinded to one another's results. ED and ES volumes (EDV and ESV) were calculated using Simpson's method and ejection fractions (EFs) determined. 

For comparison, left ventricular (LV) short axis endocardial contours were also drawn in all cases by each reader. The papillary muscles were considered to be part of the blood pool. The LV outflow tract was included up to the level of the aortic valve. LV EDV, ESV, and EF were calculated.

**Figure 3:**
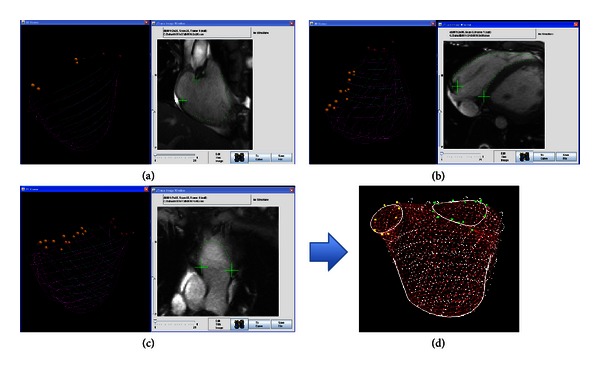
Multiplanar ED and ES data were combined to create a 3D representation of the RV that was then used to generate RV volume measurements using the PSSS method. (a)–(c) Representative sequential screen-saver images obtained during the contouring of the PSSS model. Endocardial contours of the RV free wall (purple lines) and septum (aqua lines) and anatomical landmarks such as the tricuspid valve (orange dots) and pulmonary valve (dark red dots) annuli were delineated on gray-scale images, eventually generating a 3D wire frame model of the RV. (d) This was subsequently reconstructed as a 3D triangulated mesh.

*PSSS Method.* The PSSS method was employed as the comparison standard for RV volume measurements and ejection fraction. This method has been validated for accuracy in representing not only volume but also the 3D shape of the LV and RV [11, 12]. The method accepts borders traced from any combination of imaging orientations, so that analysis of views subject to partial volume averaging can be avoided. The employment of multiple imaging orientations enables careful definition of the apex and reconstruction of the outflow tract and valve orifices. These attributes have made the PSSS method applicable for assessment of regional as well as global function and shape in congenital heart disease [16–18]. Using custom software the contours of the RV endocardium and anatomical landmarks such as the apex and valve orifices are drawn manually and used to reconstruct the RV as a triangulated mesh. Analysis using the home-grown software—while robust—is time-consuming. In its current form, this software is not sufficiently user-friendly for routine clinical use. Representative images from the PSSS method are shown in Figure 3.

The interobserver variability of PSSS analysis was previously measured in a cohort of patients with congenital heart disease whose EDV ranged from 91 to 336 mL [18]. The coefficient of variability was 4.3% for EDV, 7.7% for ESV, and 8.9% for EF. The absolute difference between observers normalized by mean values averaged 3.6 ± 2.0% for EDV, 7.3 ± 5.2% for ESV, and 11.6 ± 10.2% for EF. There was no bias in the distribution of the error. The intraclass correlation coefficients were 0.983, 0.942, and 0.796 for EDV, ESV, and EF, respectively.

For this analysis, a combination of TA, SA, HLA, and the additional orientations described in the *Axes* Section was used. However, to minimize partial volume effects, we selected only those slices where RV endocardium was distinctly visualized. The custom software does not require closed contours; this means that, for a given image, a wall segment having easily delineated endocardium could be traced, while other segments with poorly defined blood-tissue interfaces could be excluded. The number of slices used for each subject depended on the RV volume and ranged from 14 to 18. A board certified cardiologist with extensive experience in contour tracing (29 years) completed all of the contours for the PSSS analysis at ED and ES. 

### 2.4. Statistical Analysis

Interrater reliability among the three raters was estimated using the intraclass correlation coefficient (ICC) for ESV, EDV, and EF. Here, the ICC represented the proportion of variability in the measurements that was due to subject differences and not to reader differences. The higher the number is (approaching 1.0), the more reliable the data between readers are (interrater reliability). With regard to agreement, an ICC value of 0.70 was considered fair, 0.80 good, 0.90 excellent, and 1.0 perfect (no interrater variability). Systematic differences between readers in volumes calculated were tested using mixed models that accounted for differences in variances and for all covariances between measurement techniques (PSSS, TA, SA, and HLA) and cardiac phases (ED, ES). This was achieved by specifying the interaction of technique and phase as a repeated effect with patient as the subject, using an unstructured variance-covariance model. A similar but simpler model was constructed for ejection fractions (EFs), which included only effects for measurement technique. Interrater reliability for LV EDV, ESV, and EF was determined in the same way. 

Means of EDV, ESV, and EF for each of the three orientations were subsequently compared with those obtained using the PSSS method.

## 3. Results

### 3.1. Interrater Reliability (Table 2)

The ICCs for interrater reliability for RV EDV and ESV were good to excellent for all orientations. However, only the TA orientation showed excellent agreement for *both* measurements. The ICCs for RV EF were fair for the TA and HLA orientations (0.67 and 0.69, resp.) but much better for the SA orientation, which was poor (0.42). 

Bland and Altman plots for each of the reviewer pairs (R1 and R2, R1 and R3, and R2 and R3) are provided in the Figure 5 for RV EDV, RV ESV, and RV EF for transaxial (TA), short axis (SA), and horizontal long axis (HLA) views. These plots are consistent with the interrater reliability data.

The reliability coefficients were excellent for LV EDV and LV ESV and good for LV EF. 

### 3.2. Accuracy (Table 3)

**Figure 4:**
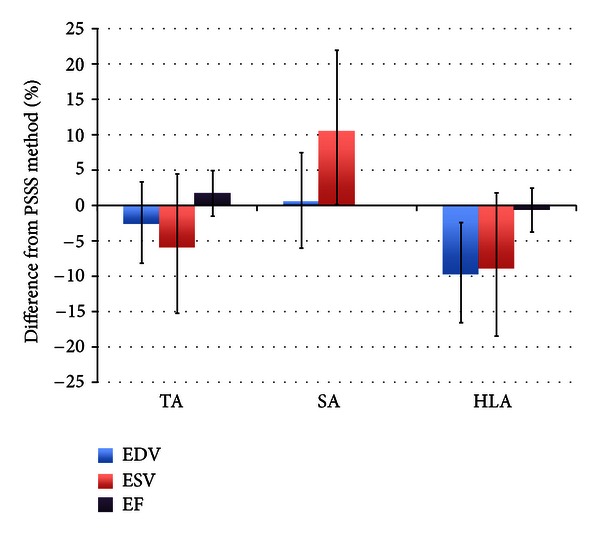
Bar graph showing the differences of reader-averaged values of right ventricular EDV, ESV, and EF obtained using Simpson's method and those obtained using the PSSS method. Error bars reflect 95% confidence intervals. Mean EF for the SA orientation was not determined due to poor interrater reliability.

**Figure 5:**
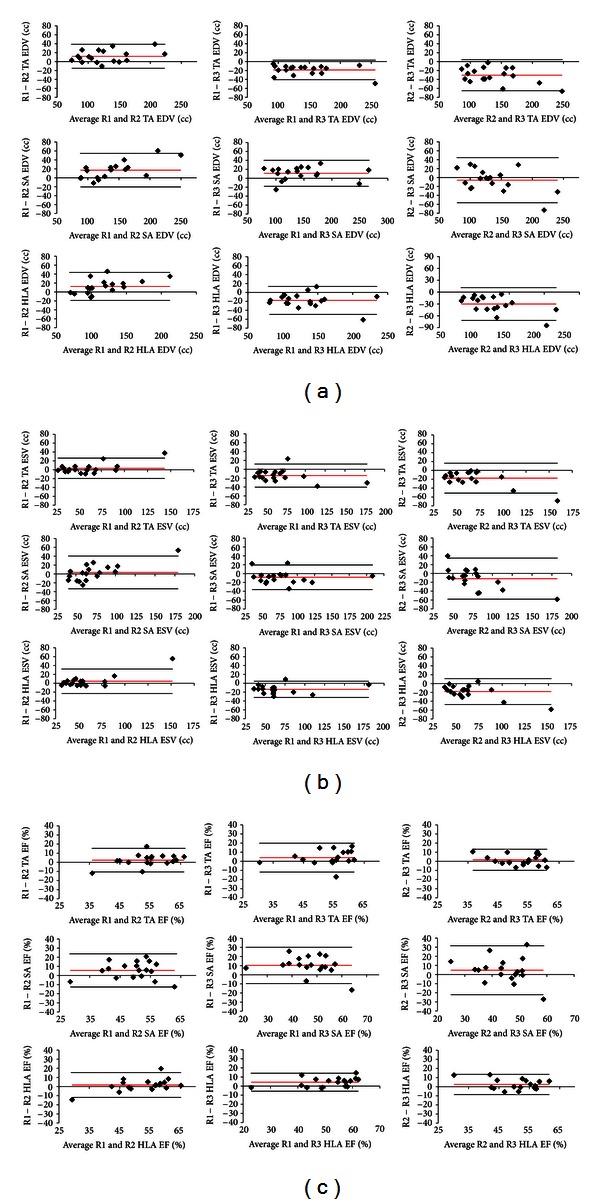
Bland and Altman plots for each of the reviewer pairs (R1 & R2, R1 & R3, and R2 & R3) are shown for (a) RV EDV, (b) RV ESV, and (c) RV EF for transaxial (TA), short axis (SA), and horizontal long axis (HLA) views. In all, 27 plots are shown. For all plots, reviewer differences are plotted against reviewer averages. The reds lines represent mean differences and the black lines indicate ±1.96 standard deviations of the differences.

The average EDV and ESV were 139.4 ± 46.9 cc (range: 82.2–245.1 cc) and 69 ± 34.8 cc (range: 30.8–174.0 cc), respectively. Since the interrater reliability of RV EDV and ESV measurements was high for all orientations, data were averaged across readers. Compared with the PSSS method, the SA orientation slightly but significantly overestimated the ESV, and the HLA orientation significantly underestimated the EDV (Figure 4). No significant differences were seen for EDV or ESV using the TA orientation. 

Of note, the EDV and ESV measurements are underestimated to a comparable extent for both TA and HLA orientations (≤3.3% difference). With the SA orientation, however, ESV measurements are overestimated to a greater degree than EDV measurements (10.0% difference). It is also noted that errors are greater for ESV measurements than for EDV measurements for all orientations and that the TA orientation has the smallest errors for both EDV and ESV measurements.

The average RV EF was 52.3 ± 9.1% (range: 29–65%). Data for TA and HLA orientations were averaged across readers, but SA EF data were not because of poor interrater reliability. For RV EF, neither TA nor HLA significantly differed from the PSSS method. 

## 4. Discussion

Although MRI is considered to be the gold standard for measuring RV volume, consensus is lacking on methodological issues such as border tracing [19]. The aim of this study is to determine which of the three orientations (TA, SA, and HLA) is best for quantitative assessment of RV chamber volume and ejection fraction using Simpson's method of slice summation. To our knowledge use of the HLA orientation for RV quantification is novel. Prior authors have demonstrated superior inter- and intraobserver reproducibility of the TA orientation over the SA orientation in both normal subjects [9] and patients with corrected Tetralogy of Fallot [10]. However, these earlier studies did not assess accuracy. 

We have found that the TA orientation is superior to both SA and HLA orientations for accurate and reliable determination of RV volumes and EF. The HLA orientation underestimates EDV, and the SA orientation both overestimates ESV and has poor interrater reliability for EF. 

It is likely that most of the variability between readers for any orientation is due to partial volume averaging. This in turn depends on the manner in which the various planes divide the RV and how they depict the interface of the RV with the blood pool. Despite the high native contrast between blood pool and myocardium on SSFP CMR, the interface may be indistinct when it is not orthogonal to an imaging slice of finite thickness. This blurring will affect not only how a contour is drawn but also which slices are included in the analysis. In general, the adverse effects of partial volume averaging may be exacerbated at end-systole where fewer slices are needed to span the chamber and the “compromised” slices receive greater weighting. (The relatively larger ESV errors support this claim.) While each of the readers in this study was experienced in using the contour tracing software, contour delineation remains challenging in specific areas of the RV for each of the three orientations. The 8 mm slice thickness used in this study may accentuate partial volume effects in these trouble regions, and a thinner slice thickness may reduce these effects. However, in designing this study we felt that it was important to use standard clinical imaging parameters to maintain clinical relevance. 

Challenging regions for the SA orientation include the tricuspid valve (TV) plane, the RVOT, the pulmonary valve (PV), and the apex [5, 16, 20]. An SA slice passing through the TV plane, for example, may simultaneously sample right atrium, main pulmonary artery, and RV cavity. Teasing out these various elements can be difficult. Occurring near the base of the heart where the RV is large, the resulting partial volume effect may lead to a substantial error in volume measurements. The tendency in our study is to overestimate ESV. Grothues et al. [20] have suggested that this basal slice effect is responsible for greater interstudy variability in measuring RV volume compared to LV volume using the SA orientation. Using the PSSS method for volume measurements, Moroseos et al. [16] have also shown for a cohort of six normal subjects that RV volume is accurately measured from the SA but that EF is inaccurate. Conversely, however, they found that for patients with transposition of the great arteries RV volume determination is inaccurate. The authors also suggest that errors occurring at the base of the heart are problematic and—like Grothues et al.—imply that greater accuracy may be achieved by considering long and short axes together.

The HLA and TA orientations offer good visualization of the TV, RV free wall, and the apex. For these orientations difficulties in contour delineation may occur near the PV and along the diaphragmatic surface of the heart where the RV inferior wall becomes essentially parallel to the imaging plane. Errors near the PV are probably small since the region in question is typically small. However, the inferior surface of the RV can be large, and the decisions of whether or not to include a slice and how to contour it may have a greater impact on the error of volume estimates. In our study there was a tendency for TA and HLA to underestimate EDV and ESV, but they did so in a consistent manner, preserving the accuracy of EF results. 

This study has limitations. Though comparable to that of prior studies, our sample size is relatively small. With regard to study execution, blurring of images from subject motion may also lead to incorrect contours. The PSSS method itself also requires manual tracing that may introduce error in the 3D analysis. Since this method benefits from a combination of long and short axes and largely avoids the partial volume averaging effects that compromise the other orientations, we believe that measurements made using this method are nevertheless the best available.

## 5. Conclusion

For comprehensive assessment of RV EDV, ESV, and EF, the TA orientation is preferred. For EF quantification, the SA orientation has poor interrater reliability and should not be used.

## Figures and Tables

**Table 1:** Patient demographics and CMR study indication.

No.	Sex	Age (y)	Height (m)	Weight (kg)	Study indication
1	M	18	1.78	68.0	Cardiomyopathy
2	M	49	1.75	81.6	Coronary artery disease
3	M	19	1.75	104.3	Preoperative assessment for noncardiac surgery (limited echo)
4	F	20	1.63	54.4	Question of aortic aneurysm
5	F	73	1.52	78.5	Aortic stenosis
6	M	36	1.63	66.7	Question of coarctation
7	F	37	1.52	65.8	Arrhythmia
8	F	50	1.65	90.7	Question of atrial septal defect
9	F	53	1.68	59.0	Question of constrictive pericarditis
10	M	19	1.93	97.5	Arrhythmia
11	M	57	1.69	81.8	Arrhythmia
12	F	21	1.78	62.1	Chest pain and syncope
13	M	48	1.78	124.7	Cardiomyopathy
14	M	67	1.80	85.3	Coronary artery disease
15	F	39	1.57	63.5	Question of patent ductus arteriosus
16	M	48	1.83	139.3	Chest pain
17	F	68	1.57	117.9	Arrhythmia
18	F	81	1.63	70.8	Mitral valve disease
19	F	57	1.52	64.4	Coronary artery disease
20	M	44	1.70	61.2	Known pseudocoarctation

**Table 2:** Intraclass correlation coefficients for interrater reliability.

Axis	EDV	ESV	EF
RV-SA	0.92	0.82	0.42
RV-TA	0.95	0.92	0.67
RV-HLA	0.85	0.93	0.69
LV-SA	0.98	0.96	0.84

EDV: end-diastolic volume, EF: ejection fraction, ESV: end-systolic volume, HLA: horizontal long axis, LV: left ventricle, RV: right ventricle, SA: short axis, and TA: transaxial.

**Table 3:** Percent difference of mean data with PSSS method.

Axis	EDV	ESV	EF
RV-SA	0.5 (−6.0 to 7.5)	10.5 (0.1 to 21.9)	—
RV-TA	−2.6 (−8.2 to 3.3)	−5.9 (−15.2 to 4.5)	1.7 (−1.5 to 4.9)
RV-HLA	−9.8 (−16.6 to −2.4)	−8.9 (−18.5 to 1.8)	−0.7 (−3.8 to 2.5)

Values are in percent. 95% confidence intervals are given in parentheses. EDV: end-diastolic volume, EF: ejection fraction, ESV: end-systolic volume, HLA: horizontal long axis, LV: left ventricle, RV: right ventricle, SA: short axis, and TA: transaxial. Mean EF for RV-SA was not determined due to the poor interrater reliability.
